# Comparison of vertical ground reaction forces during overground and treadmill running. A validation study

**DOI:** 10.1186/1471-2474-13-235

**Published:** 2012-11-27

**Authors:** Bas Kluitenberg, Steef W Bredeweg, Sjouke Zijlstra, Wiebren Zijlstra, Ida Buist

**Affiliations:** 1 Center for Sports Medicine, University Medical Center Groningen, Hanzeplein 1, Groningen, GZ, 9713, The Netherlands; 2 Center for Human Movement Sciences, University Medical Center Groningen, P.O. Box 196, Groningen, AD, 9700, The Netherlands; 3 Institute of Movement and Sport Gerontology, German Sport University Cologne, Cologne, Germany

**Keywords:** Running, Kinetics, Biomechanics, Validity, Overuse injuries

## Abstract

**Background:**

One major drawback in measuring ground-reaction forces during running is that it is time consuming to get representative ground-reaction force (GRF) values with a traditional force platform. An instrumented force measuring treadmill can overcome the shortcomings inherent to overground testing. The purpose of the current study was to determine the validity of an instrumented force measuring treadmill for measuring vertical ground-reaction force parameters during running.

**Methods:**

Vertical ground-reaction forces of experienced runners (12 male, 12 female) were obtained during overground and treadmill running at slow, preferred and fast self-selected running speeds. For each runner, 7 mean vertical ground-reaction force parameters of the right leg were calculated based on five successful overground steps and 30 seconds of treadmill running data. Intraclass correlations (ICC_(3,1)_) and ratio limits of agreement (RLOA) were used for further analysis.

**Results:**

Qualitatively, the overground and treadmill ground-reaction force curves for heelstrike runners and non-heelstrike runners were very similar. Quantitatively, the time-related parameters and active peak showed excellent agreement (ICCs between 0.76 and 0.95, RLOA between 5.7% and 15.5%). Impact peak showed modest agreement (ICCs between 0.71 and 0.76, RLOA between 19.9% and 28.8%). The maximal and average loading-rate showed modest to excellent ICCs (between 0.70 and 0.89), but RLOA were higher (between 34.3% and 45.4%).

**Conclusions:**

The results of this study demonstrated that the treadmill is a moderate to highly valid tool for the assessment of vertical ground-reaction forces during running for runners who showed a consistent landing strategy during overground and treadmill running. The high stride-to-stride variance during both overground and treadmill running demonstrates the importance of measuring sufficient steps for representative ground-reaction force values. Therefore, an instrumented treadmill seems to be suitable for measuring representative vertical ground-reaction forces during running.

## Background

One major drawback in measuring ground-reaction forces during running is that it is time consuming to get representative ground-reaction force (GRF) values with a traditional force platform. A single force platform is only capable of measuring GRFs of one single stance phase per trial [1,2]. Therefore, multiple force platforms are necessary for measuring consecutive steps which is space consuming and expensive. The limited length of a runway, also makes it difficult to simulate natural running at a constant speed in a laboratory situation [3]. For detection of small differences in GRFs during running, however, it is important to record sufficient steps during a stable running pattern [4]. An instrumented treadmill capable of measuring GRFs can overcome the limitations inherent to overground GRF testing during running at a short runway. With an instrumented treadmill it is possible to measure GRFs of multiple steps during one trial without interruptions in running speed, resulting in a more stable running pattern during the measurements [3].

**Figure 1:**
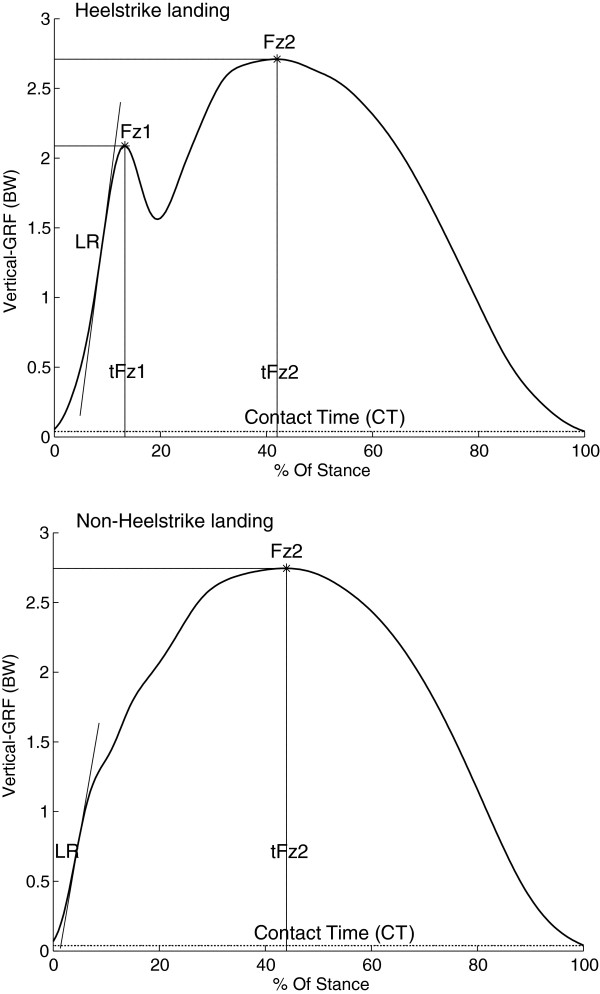
**Outcome measures in a typical vertical ground-reaction force (GRF) curve for a heelstrike runner and a non-heelstrike runner.** Figure is created from personal data.

In running, most runners make first ground contact with the posterior part of the foot, this is called heelstrike running. This running style results in a typical vertical GRF force-time curve that is characterized by two peaks, the impact peak and the active peak, as depicted in Figure 1. Magnitude of the impact peak is speed dependent and occurs during the first 10% of stance (10-30ms) [5]. The active peak is reached approximately during mid-stance and can last up to 200ms. The absence of a separate impact peak in the force-time curve is typical for a non-heelstrike runner, as depicted in Figure 1[6]. Besides a vertical component, GRFs also have an anterior-posterior and medio-lateral component. During running, the anterior-posterior force component shows a typical braking and propulsive phase while the medio-lateral force component is characterized by more variability [7]. Compared to the vertical GRF component, anterior-posterior and medio-lateral forces are small [7].

An underlying assumption when using a treadmill for running analysis is that running on a treadmill is similar to overground running. A comparison of spatio-temporal variables during overground and treadmill running was made in several studies. During treadmill running, runners tend to run with a shortened stride length and an increased stride rate [3,8,9]. Despite of these spatio-temporal differences, only small differences in knee flexion and a more flattened landing style during treadmill running were observed [3,10]. An overground-treadmill comparison with respect to GRFs was made in only two studies [1,3]. No systematic errors or extraordinary differences in vertical GRFs were found. Impact peaks and loading rates, however, have not been studied in these previous studies.

The purpose of this study was to determine the validity of a custom made instrumented force measuring treadmill to measure vertical GRF parameters during running. Validation of the treadmill was performed by comparing overground and treadmill measured vertical GRF parameters during running.

## Methods

### Participants

Twenty-four experienced runners (12 male, 12 female) between 18 and 35 years old participated in this study. The runners were voluntarily recruited by contacting two local track and field clubs. The criteria for inclusion in this study included a minimal training frequency of two times a week for at least a period of one year. Runners who reported an injury at time of measurement were excluded. Both heelstrike and non-heelstrike runners were allowed to participate in this study. All participants signed an informed consent before measurements started. The study was approved by the Medical Ethical Committee at the University Medical Center Groningen, The Netherlands; M12.112668.

### Overground measurements

During the overground measurements, GRFs were measured at three different individual speed conditions. Participants were instructed to run at their preferred speed (running speed for a normal endurance run), slower speed (running speed during a warming-up), and a faster speed (10km race speed) respectively. GRFs were collected with a force platform (0.60m x 0.40m) which was mounted in the middle of a 17.5m long runway. The sample frequency of the force platform was set at 1000Hz. Running speed was monitored with two pairs of photocells placed 2.5m before and after the force platform.

Before the actual overground measurements started, the participants performed several accommodation runs. During these accommodation runs, the exact start position for the measurements was determined. The start position was based on the position of foot placement at the force platform. Foot strike of the right foot should be completely at the force platform without an alteration in running pattern. An alteration can indicate aiming for the force platform, which modifies the GRF pattern [11]. Position of foot placement and running pattern were evaluated on sight. When participants were able to run several trials at the same speed, while landing with the right foot completely placed at the force platform, without visible alterations in running pattern, the actual measurements started. Since the participants were tested at three different speed conditions, accommodation runs were performed for each speed condition (preferred, slow and fast). The accommodation runs for the preferred speed were combined with a short warming-up and took longer (approximately 10 min), where the subsequent accommodation runs took approximately 5 minutes.

During the actual measurements, GRF data were captured until five clean strikes of the right leg within a 5% speed range were recorded for all speed conditions. Trials with visible alterations in running pattern were not included in these five clean strikes. Afterwards, the mean running speed of the five steps was calculated for each speed condition.

### Treadmill measurements

**Figure 2:**
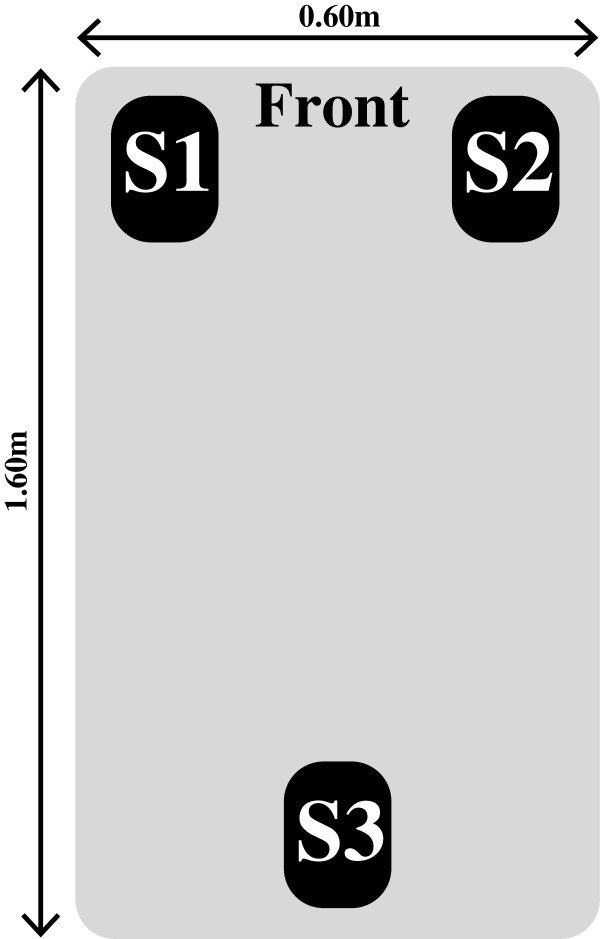
Positioning of the three strain gage force transducers S1, S2 and S3.

In this study, an instrumented treadmill (Entred, Forcelink, Culemborg, The Netherlands) with a running surface of 1.60m by 0.60m that was driven by a 1.8 kW motor was used to measure vertical GRFs during running. The treadmill was equipped with three strain gage force transducers (ACB-500kg, Vishay Revere Transducers, Breda, The Netherlands) which were connected to bridge amplifiers. The force transducers were mounted on a stiff plate which was enforced with a non-deformable frame and were positioned as shown in Figure 2. The signals from the amplifiers were sampled at 1000 Hz, digitized into a 16-bit signal by an AD converter (PCI-6220, National Instruments, Austin, TX, USA) and were connected to a computer.

Before the treadmill measurements started, participants started with an accommodation run of 10 minutes at 10 km·h^-1^. After this accommodation period, participants were tested at three different individual speed conditions (slow, preferred and fast). Treadmill speed was matched to the average overground running speed for each speed condition because GRF parameters are speed dependent [7]. The three speed conditions lasted three minutes and were offered in random order. GRFs were recorded during the last 30 seconds of each speed condition. When treadmill measurements were finished, participants were given the opportunity for cooling-down at the treadmill. All measurements were conducted while participants were running in their personal running shoes.

### Data analysis

Vertical force data from both the force platform and the treadmill were processed using custom programs written in MATLAB R2010a (The MathWorks, Inc, Natick, MA). All steps which were recorded during the treadmill measurement were entered into the analysis. A 13-point moving average low-pass filter with a cut-off frequency of 33.3Hz was used to filter the GRF data that was recorded during the overground and treadmill measurements. Foot strikes in the overground and treadmill data were detected with a threshold of 30 Newton for impact and toe-off phase. Outcome measures for all right foot steps were identified, as described in Table 1. For each speed condition outcome measures of each participant were averaged. A distinction between heelstrike and non-heelstrike landing patterns was made based on the existence of an impact peak Fz1. Peak values Fz1 and Fz2 and the loading-rate were normalized to bodyweight.

**Table 1:** **Definition of outcome measures, as displayed in Figure**1

**Outcome measure**	**Description**
Fz1	Local maximum in the vertical GRF data, normalized to body weight (BW).
Fz2	Maximum value in the vertical GRF data, normalized to BW.
LR	The steepest part of the vertical GRF curve, from stance to impact peak. Expressed in BW/s.
ALR	Average loading rate, the slope of the line from 20% to 80% of Fz1. Expressed in BW/s.
tFz1	Time from heelstrike to Fz1 in ms.
tFz2	Time from heelstrike to Fz2 in ms.
CT	Contact time, from heelstrike to toe-off in ms.

Outcome measures for the overground and treadmill data were identified with the same routine. Foot strikes were detected with a threshold of 30 Newton for both heelstrike and toe-off.

### Statistical analysis

A within-subject repeated measures design was used to determine the validity of the instrumented treadmill for measuring vertical GRF parameters during running. Therefore, a two-way mixed-effects, consistency, single measure intraclass correlation coefficient (ICC_(3,1)_) model was used to examine the agreement between overground and treadmill measured GRF-parameters. Interpretation of the intraclass coefficients were as follows: poor (0 – 0.39), modest (0.4 – 0.74), or excellent (0.75 – 1) [12]. ICCs were calculated by using SPSS (SPSS inc. Version 18.0, Chicago, IL, U.S.A.). Besides the intraclass correlations, Bland-Altman plots were used to examine the agreement between overground and treadmill measurements [13]. These plots were made for each outcome measure and each speed condition. The limits of agreement (LOA) were calculated (mean difference +/− 1.96 times the standard deviation of the difference). Also ratio limits of agreement (RLOA) were calculated to express the LOA as percentage of the mean overground-treadmill value. The upper and lower LOA and the RLOA provide insight into how much random variation may be influencing the measurements.

## Results

Ground-reaction force (GRF) parameters of a different landing strategy cannot be compared, therefore only GRF parameters of participants who showed a consistent landing strategy during overground and treadmill running within a speed condition were examined. During overground running at preferred speed, 19 participants showed a heelstrike (HS) landing, while 16 of these runners showed a HS landing during treadmill running. This shows that 82.4% of the runners used a similar landing strategy during treadmill running at preferred speed. Results for the two other speeds can be found in Table 2.

**Table 2:** Overground landing strategy compared to treadmill landing strategy, displayed as number of persons and corresponding percentages of runners who showed a consistent landing strategy

	**Heelstrike landing**			**Non-heelstrike landing**		
	**Overground**	**Treadmill**	**Consistency**	**Overground**	**Treadmill**	**Consistency**
Slow	17	14	82.4%	7	5	71.4%
Preferred	19	16	84.2%	5	5	100.0%
Fast	12	12	100.0%	12	6	50.0%

**Figure 3:**
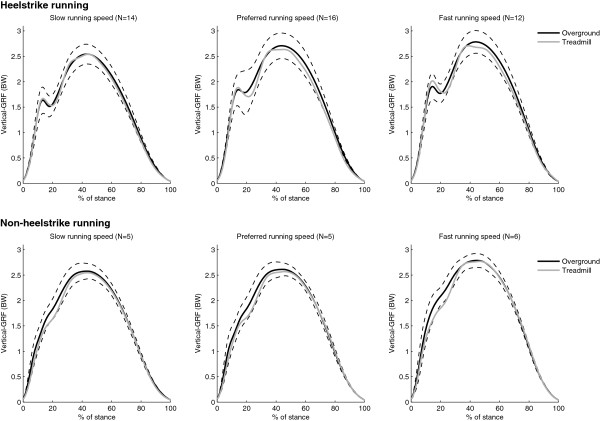
**Average GRF plots from all runners for overground (mean, solid black line; ± SD, dotted black lines) and treadmill running (mean, solid grey line) at slow, preferred and fast running speed for heelstrike and non-heelstrike runners.** Forces are in body weight (BW).

Qualitatively, the overground and treadmill GRF curves for both HS and NHS running at slow, preferred and fast running speeds, were very similar, as can be seen in Figure 3. In Table 3 a quantitative evaluation of the vertical GRF-parameters of both HS and NHS runners can be found. The levels of agreement between overground and treadmill running for the time related variables (tFz1, tFz2 and CT) were excellent (ICCs between 0.76 and 0.95 and RLOAs between 5.7% and 15.5%). Also the active peak (Fz2) measured with both devices showed excellent agreement (ICCs between 0.77 and 0.89, RLOAs between 7.8% and 9.9%). Modest agreement was found for the impact peak, Fz1 (ICCs between 0.71 and 0.76, RLOAs between 19.9% and 28.8%). Maximal loading rate (LR) and average loading rate (ALR) also showed modest to excellent intraclass correlations (ICCs between 0.70 and 0.89), however the ratio limits of agreement were higher (RLOA values between 34.3% and 45.4%).

**Table 3:** Outcome measures for overground and treadmill running

		**OG mean ± SD**	**TM mean ± SD**	**ICC**_**(3,1)**_**(95%CI)**	**Mean diff (LOA) diff (lowLim, upLim)**	**RLOA (%)**
*Fz1* (*BW*)						
HS	Slow	1.67 ± 0.26	1.70 ± 0.23	0.74 (0.37, 0.91)	0.03 (−0.32, 0.38)	20.8
	Preferred	1.94 ± 0.45	1.93 ± 0.30	0.71 (0.35, 0.89)	−0.01 (−0.57, 0.55)	28.8
	Fast	1.94 ± 0.25	2.06 ± 0.32	0.76 (0.35, 0.92)	0.12 ( −0.28, 0.52)	19.9
*Fz2* (*BW*)						
HS	Slow	2.54 ± 0.20	2.53 ± 0.18	0.77 (0.49, 0.91)	−0.02 (−0.27, 0.23)	9.9
	Preferred	2.70 ± 0.26	2.65 ± 0.25	0.89 (0.76, 0.96)	−0.03 (−0.25, 0.17)	7.9
	Fast	2.77 ± 0.24	2.70 ± 0.22	0.86 (0.67, 0.95)	−0.06 (−0.27, 0.15)	7.8
NHS	Slow	2.56 ± 0.17	2.55 ± 0.20			
	Preferred	2.61 ± 0.15	2.58 ± 0.13			
	Fast	2.79 ± 0.15	2.78 ± 0.20			
*LR* (*BW*/*s*)						
HS	Slow	81.11 ± 25.62	87.28 ± 23.39	0.76 (0.47, 0.90)	3.25 (−28.62, 35.12)	39.9
	Preferred	95.34 ± 26.67	105. 33 ± 25.08	0.80 (0.57, 0.91)	6.11 (−26.21, 38.42)	34.3
	Fast	104.40 ± 29.29	118.08 ± 33.73	0.70 (0.36, 0.88)	7.17 (−37.69, 52.02)	42.7
NHS	Slow	70.03 ± 14.68	65.09 ± 13.74			
	Preferred	77.00 ± 22.35	74.25 ± 16.47			
	Fast	95.81 ± 26.02	87.41 ± 18.74			
*ALR* (*BW*/*s*)						
HS	Slow	68.89 ± 20.26	73.92 ± 20.22	0.84 (0.63, 0.93)	2.98 (−24.74, 30.70)	45.3
	Preferred	82.14 ± 21.38	88.70 ± 20.75	0.89 (0.74, 0.95)	3.60 (−23.01, 30.21)	36.4
	Fast	90.70 ± 23.66	100.77 ± 29.10	0.86 (0.67, 0.95)	4.08 (−31.26, 39.42)	45.4
NHS	Slow	33.96 ± 6.07	31.21 ± 5.01			
	Preferred	47.09 ± 22.92	33.78 ± 4.20			
	Fast	43.63 ± 13.89	36.00 ± 4.20			
*tFz1* (*ms*)						
HS	Slow	35 ± 4.08	35 ± 4.86	0.76 (0.40, 0.92)	0.0 ( −5.4, 5.4)	15.5
	Preferred	34 ± 4.42	34 ± 3.35	0.82 (0.56, 0.93)	0.3 ( −4.4, 5.0)	13.8
	Fast	32 ± 5.00	33 ± 4.88	0.87 (0.61, 0.96)	0.6 ( −3.7, 4.8)	13.0
*tFz2* (*ms*)						
HS	Slow	112 ± 13.55	109 ± 10.22	0.84 (0.63, 0.94)	−1.8 (−15.4, 11.8)	12.6
	Preferred	102 ± 13.28	100 ± 12.19	0.94 (0.85, 0.97)	−1.6 (−10.1, 6.8)	8.5
	Fast	99 ± 10.00	96 ± 11.47	0.87 (0.68, 0.95)	−3.0 (−13.5, 7.5)	11.0
NHS	Slow	102 ± 13.00	103 ± 15.00			
	Preferred	99 ± 12.00	98 ± 11.00			
	Fast	92 ± 80 0	91 ± 10.00			
*CT* (*ms*)						
HS	Slow	258 ± 22.00	254 ± 21.13	0.92 (0.80, 0.97)	−4.0 (−21.4, 13.4)	6.9
	Preferred	232 ± 23.34	232 ± 20.49	0.92 (0.82, 0.97)	−2.0 (−17.2, 13.2)	6.6
	Fast	223 ± 21.00	220 ± 21.14	0.95 (0.87, 0.98)	−3.3 (−15.6, 9.1)	5.7
NHS	Slow	240 ± 17.00	237 ± 19.00			
	Preferred	229 ± 12.00	222 ± 12.00			
	Fast	213 ± 12.00	206 ± 12.00			

Intraclass correlations, mean-differences with limits of agreement (LOA), and ratio limits of agreement (RLOA) were reported. Both HS and NHS runners were taken into account in the statistical analysis. Number of participants: HS (slow: N=14, preferred: N=16, fast: N=12), NHS (slow: N=5, preferred: N=5, fast: N=6).

HS: Heelstrike-runner, NHS: Non-Heelstrike-runner, CI: Confidence Interval, LOA: Limit of Agreement, RLOA: Ratio Limit of Agreement, OG: Overground, TM: Treadmill, BW: Body Weight.

Slow running speed: HS runners: 11.0 ± 1.3 km·h^-1^, NHS runners: 10.9 ± 1.5 km·h^-1^.

Preferred running speed: HS runners: 12.7 ± 1.6 km·h^-1^, NHS runners: 11.8 ± 1.5 km·h^-1^.

Fast running speed: HS runners: 14.1 ± 2.0 km·h^-1^, NHS runners: 13.9 ± 1.9 km·h^-1^.

## Discussion

The instrumented treadmill is capable of measuring vertical ground-reaction forces (GRFs) during running and seems to be a usable tool for simulating overground running kinetics. The results of this study demonstrated that the instrumented treadmill is a highly valid tool for the assessment of the vertical GRF parameters: tFz1, tFz2, CT and Fz2 and moderately valid for the assessment of Fz1, LR and ALR for runners who showed a consistent landing strategy during overground and treadmill running. A qualitative evaluation of the overground and treadmill vertical GRF curves as shown in Figure 3, demonstrated that the vertical GRFs for both the heelstrike (HS) runners and the non-heelstrike (NHS) runners were similar during overground and treadmill running. The excellent intraclass correlations and low limits of agreement for contact time (CT), time to impact peak force (tFz1) and time to the active peak (tFz2) reflect this qualitative similarity. After all, these parameters show that the timing of peak values in the vertical GRF curve is not different for overground and treadmill running. The qualitative similarity of these GRF curves was also observed in other studies [1,3]. In the current study, the overground and treadmill measured active peak (Fz2) showed no noteworthy differences. This is in accordance with the results of Riley et al., who also compared overground and treadmill running kinetics in a group of 20 runners [3]. Overground and treadmill measured impact peaks (Fz1), maximal loading rates (LR) and average loading rates (ALR), showed less consistent results with modest to excellent intraclass correlations and wider limits of agreement. To our knowledge this study is the first to compare overground and treadmill measured impact peaks and loading rates during running, therefore it is not possible to evaluate these results with previous studies.

For an overground-treadmill comparison with respect to vertical GRF parameters, a consistent landing strategy during both running conditions (overground and treadmill) is required. While most runners showed a consistent landing strategy during overground and treadmill running, some runners switched to another landing strategy. During slow and preferred running speed, these inconsistent runners mostly switched from an overground HS landing to a NHS landing during treadmill running. Considering that this behavior is in line with the more flattened landing style as observed in a previous study [14], it is likely that these inconsistencies in landing strategy are the result of accommodation to treadmill running. At fast self selected speed, however, the inconsistent runners switched from a NHS to a HS landing during treadmill running. These differences in landing strategy may indicate overground and treadmill differences in anterior-posterior GRFs which were not compared in the current study. The results of this study demonstrated that the inconsistencies in landing strategy are smallest during running at preferred speed. Therefore, to maximize certainty, it can be recommended to determine landing strategy with a treadmill measurement at preferred running speed.

The use of a treadmill in a research setting has been subject of much debate. Several factors are mentioned which may cause biomechanical differences between overground and treadmill running [9]. First, non-mechanical factors as accommodation to the changed visual and auditory surroundings or fear during treadmill running may result in differences between overground and treadmill running biomechanics [15]. Second, differences in air resistance may have an effect on treadmill running form [16]. The effects of air resistance on running kinematics, however, will only be visible during running at high speeds [17]. Third, intra-stride belt speed variations, due to an energy exchange between the treadmill and the runner, can cause differences in running kinematics compared to overground running. In particular low powered treadmills are more sensitive for opposite forces acting on the belt during running, resulting in larger belt speed variations. These variations in belt speed may lead to biomechanical differences during treadmill running when compared to overground running [15]. Fourth, during running, leg stiffness is adjusted to the stiffness of the running surface [18]. Adjusting leg stiffness results in subtle changes in the kinematics of the lower extremity [19]. Therefore, differences in running surface may lead to biomechanical differences when comparing overground and treadmill running.

Several studies compared overground and treadmill running biomechanics [3,8,14]. Even though runners tend to run with a shortened stride length and an increased stride rate during treadmill running [3,8,9], overground and treadmill running kinematics are remarkably similar [3,9,14]. Only small differences in knee and ankle joint kinematics were reported. Nigg et al. observed a more flattened landing style during treadmill running [14]. Riley et al. did not find differences in ankle joint kinematics, but did find differences in minimal and maximal knee flexion [3]. Maximal knee flexion was lower and minimal flexion was higher during treadmill running, which could be a result of the observed decrease in flight phase and higher stride rate [3]. Thus, despite the theoretical factors which may influence treadmill running biomechanics, only small differences in overground and treadmill kinematics were observed. In the current study, also no significant differences in GRF parameters between overground and treadmill running were found. These findings are in line with previous studies where overground and treadmill running kinetics were compared [1,3]. The between person variance in Fz1, LR and ALR during both overground and treadmill running was high, as indicated by the high standard deviations for these parameters. Stride-to-stride variance for these parameters was also high, which demonstrates the importance of measuring sufficient steps for representative GRF values. This is especially important for detecting small differences between different conditions or persons [20]. Because a treadmill makes it possible to measure multiple steps during one test trial, it can be argued that a treadmill measurement is more suitable for detecting small differences in vertical GRFs during running. However, this assumption was not assessed in the current study.

Since the treadmill used in the current study only is capable of measuring vertical GRFs it cannot be used to assess joint kinetics using the standard inverse dynamics methodology, because anterior-posterior and medio-lateral GRFs are also needed for these calculations. It should also be noted that the inconsistencies in landing strategy may indicate differences in anterior-posterior GRFs between overground and treadmill running. Furthermore, this instrumented treadmill would have limited usefulness for walking studies, because the double support phase in walking cannot be measured directly. For measuring GRFs during walking, an instrumented split-belt treadmill may be more convenient.

A limitation of this study was that participants first performed the overground measurements after which the treadmill measurements started. Due to this fixed order of the measurements, fatigue may have influenced the later treadmill measurements [21]. Nevertheless, this influence is expected to be low, since all participants were experienced runners who did not have to deliver a maximal performance and participants did not show signs of exaggerated fatigue during the measurements.

## Conclusions

The results of this study demonstrated the treadmill is a moderate to highly valid tool for the assessment of vertical GRFs during running for runners who showed a consistent landing strategy during overground and treadmill running. Therefore, an instrumented treadmill can be used to measure vertical GRF parameters which correspond to normal overground values during running.

In a future study, the treadmill can be used to measure vertical GRF parameters in a large group of runners, for instance to identify possible kinetic risk-factors for running related injuries prospectively.

## Abbreviations

GRF: Ground-reaction force; HS: Heelstrike; NHS: Non-heelstrike; Fz1: Impact peak; Fz2: Active peak; LR: Loading Rate; ALR: Average Loading Rate; CT: Contact Time; tFz1: Time to impact peak; tFz2: Time to active peak; BW: Body Weight; ICC: Intraclass correlation; LOA: Limit of Agreement; RLOA: Ratio Limit of Agreement.

## Competing interests

In this study, an instrumented treadmill was used. The research group had no financial or other interest in the treadmill product or distributor of the treadmill. The project was not dependent on external financial assistance and the authors declare that they have no competing interests.

## Authors’ contributions

SB, SZ, WZ and IB provided advice on the study design. BK recruited the participants, was responsible for the data acquisition/analysis and wrote the article. WZ provided advice on the data analysis. SB, SZ, WZ and IB contributed to the content of the article. All authors read and approved the final manuscript.

## Pre-publication history

The pre-publication history for this paper can be accessed here:

http://www.biomedcentral.com/1471-2474/13/235/prepub
